# Genetic mapping high protein content QTL from soybean ‘Nanxiadou 25’ and candidate gene analysis

**DOI:** 10.1186/s12870-021-03176-2

**Published:** 2021-08-20

**Authors:** Jia Wang, Lin Mao, Zhaoqiong Zeng, Xiaobo Yu, Jianqiu Lian, Jun Feng, Wenying Yang, Jiangang An, Haiying Wu, Mingrong Zhang, Liezhao Liu

**Affiliations:** 1 Nanchong Academy of Agricultural Sciences, Nanchong, 637000 Sichuan China; 2 Southwest University, Chongqing, 400715 China

**Keywords:** Soybean, Seed protein content, Quantitative trait loci (QTL), QTL-seq, RNA-seq

## Abstract

**Background:**

Soybean is a globally important legume crop that provides a primary source of high-quality vegetable protein and oil. Seed protein content (SPC) is a valuable quality trait controlled by multiple genes in soybean.

**Results:**

In this study, we performed quantitative trait loci (QTL) mapping, QTL-seq, and RNA sequencing (RNA-seq) to reveal the genes controlling protein content in the soybean by using the high protein content variety Nanxiadou 25. A total of 50 QTL for SPC distributed on 14 chromosomes except chromosomes 4, 12, 14, 17, 18, and 19 were identified by QTL mapping using 178 recombinant inbred lines (RILs). Among these QTL, the major QTL q*SPC*_20–1 and q*SPC*_20–2 on chromosome 20 were repeatedly detected across six tested environments, corresponding to the location of the major QTL detected using whole-genome sequencing-based QTL-seq. 329 candidate DEGs were obtained within the QTL region of q*SPC*_20–1 and q*SPC*_20–2 via gene expression profile analysis. Nine of which were associated with SPC, potentially representing candidate genes. Clone sequencing results showed that different single nucleotide polymorphisms (SNPs) and indels between high and low protein genotypes in *Glyma.20G088000* and *Glyma.16G066600* may be the cause of changes in this trait.

**Conclusions:**

These results provide the basis for research on candidate genes and marker-assisted selection (MAS) in soybean breeding for seed protein content.

**Supplementary Information:**

The online version contains supplementary material available at 10.1186/s12870-021-03176-2.

## Background

With an average composition of approximately 40% protein, soybean (*G. max* (L.) Merr.) is the most important source of vegetable protein, accounting for 71% of dietary consumption and this proportion is still rising year by year [1]. There is a wide variation of seed protein content (SPC) in soybean. According to the database of soybean germplasm resources of USDA (https://npgsweb.ars-grin.gov/), the SPC of cultivated soybean is 31.7–57.9%, and that of wild soybean is 35.5–56.9%, suggesting that there is great potential for genetic improvement of soybean SPC. While increasing SPC of soybean cultivars has been a major objective of many soybean breeding programs for decades, the strong negative correlations of SPC with seed oil content and seed yield improve three traits simultaneously a challenging task using conventional breeding [2–4]. Most of the soybean grown worldwide is commodity soybean for which farmers are paid by weight and not a composition. Consequently, in cultivar development, breeders generally select for the best seed yield potential with little attention to seed protein or oil content [5]. Therefore, high yield or high oil content is generally pursued in large-scale soybean varieties. There are few large-scale soybean varieties with high protein content, and even fewer varieties with protein content more than 50%. Considering people’s demand for vegetable protein and the economic benefits of farmers, identification of molecular markers associated with quantitative trait loci (QTL) controlling seed yield, seed protein, and oil content is a necessary prerequisite for breaking the negative correlations between these traits.

Classical quantitative genetic analysis shows that SPC of soybean is a quantitative character with additive effect and governed by multiple genetic loci subject to genotype×environment interactions [4, 6–8]. Linkage analysis is a useful approach for dissecting complex traits at the molecular genetics level in plants. Since Diers et al. (1992) first used linkage analysis to discover a major QTL connected to soybean protein and oil content on the chromosome (Chr.) 20 [9], a large number of QTL related to SPC have been reported in succession. Before this study, 241 QTL loci with SPC have been recorded in the soybase database (http://soybase.org/), involving every chromosome in the biparental population. Furthermore, genome-wide association studies (GWAS) have been widely used in soybean complex traits studies, including SPC [5, 10–15]. The Soybase website has listed 62 QTL linked to protein content collected from 2015 to 2018, involving every chromosome in the natural population. Although a large number of soybean protein content related QTL were detected on each chromosome, most of them were not detected frequently, and only 57 of these QTL were verified [16]. The summary results showed that no matter linkage analysis or GWAS, the frequency of QTL related to protein content was the highest on chromosome 20 (linkage group I, LG I), followed by chromosome 15 (linkage group E, LG E). Many candidate genes were analyzed in the confidence intervals of these QTL, especially on chromosome 20. Bolon et al. (2010) found that 13 genes displaying significant seed transcript accumulation differences between NILs were identified that mapped to the 8.4 Mbp QTL region on LG I by AffymetrixÂ® Soy GeneChip and high-throughput IlluminaÂ® whole transcriptome sequencing platforms [17]. Hwang et al. (2014) further reduced the QTL interval to 2.4 Mbp through association analysis [4]. In this region, the number of candidate genes was reduced to six (*Glyma20g19680*, *Glyma20g21030*, *Glyma20g21080*, and three unrelated genes). The above candidate genes have a certain distance in the physical map, but if these gene development markers are used for genetic analysis, the distance in the genetic map may be very close. Therefore, there may be very important candidate genes in this region to be further verified.

Bulked segregant analysis (BSA) is a simple and rapid method for target gene mapping [18]. According to different group materials and experimental designs, BSA can be divided into the following types: (1) QTL-seq is suitable for quality traits and quantitative traits with significant major genes [19]; (2) MutMap is suitable for mutant lines analysis [20]; (3) Mutmap (also called MutMap+) is suitable for early lethal or non-heterozygous mutants [21]; (4) Mutmap-gap is suitable for traits whose target gene is not on the reference genome of species [22]. In recent years, BSA has been widely used in the genetic mapping of important traits, such as *A. thaliana* [23, 24], rice [19, 20], and maize [25]. The mapping method based on BSA has also been widely used in soybean related traits study, including plant height [26, 27], flowering time [28], phytophthora resistance [29], and cotyledon color of seed [30]. Compared to traditional QTL mapping methods, BSA only requires consideration of a few extreme individuals in the population rather than the entire population, simplifying the sequencing process and significantly reducing the cost of sequencing and analysis [31]. With the development of DNA sequencing technology, next-generation sequencing (NGS)-based BSA approaches dramatically accelerated and improved the identification process of causal genes [32].

Nanxiadou 25 bred from offspring of ^60^Coγ radiation-induced mutation of Rongxiandongdou through several years’pedigree selections, is a high protein soybean variety widely cultivated in southwest China. The SPC of the variety is 50.1%, which has good shading tolerance, strong lodging tolerance, resistance to Soybean Mosaic Virus Strain SC3 and SC7, and other excellent characteristics, and is suitable for intercropping with maize. In this study, we used a mapping population developed from a cross between ‘Nanxiadou 25’ and ‘Tongdou 11’ and an F_2_ segregant population developed from a cross between ‘Nanxiadou 25’ and ‘Rongxiandongdou’. In this study, we combined QTL mapping, QTL-seq, whole-genome resequencing (WGRS), and RNA-seq to analyze the QTL and candidate genes of soybean protein content, in order to widen the genetic base in soybean towards crop improvement. The objectives of this study were to identify stable QTL for SPC and to mining potential candidate genes located within associated genomic regions, and to develop corresponding molecular markers, which ultimately may be used to facilitate the development of high-protein soybean lines using marker-assisted selection (MAS).

## Results

### Phenotypic characteristics of SPC

**Fig. 1:**
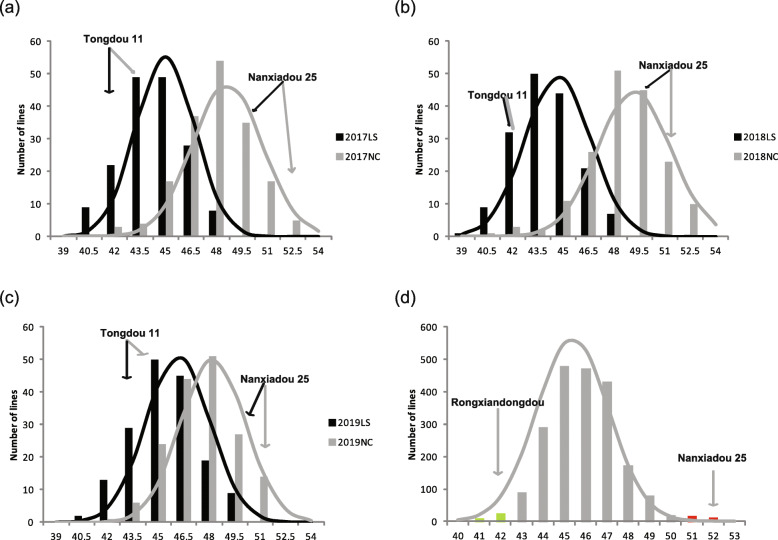
Frequency distribution of SPC among RILs and F_2_ population along with two parents. (a-c) Represents the distribution of SPC of RILs population in the environment of 2017LS, 2017NC, 2018LS, 2018NC, 2019LS, and 2019NC, respectively; (d) Represent the distribution of SPC among 2097 F_2_ plants in the environments of 2019NC. Green and red bars represent LP-bulk and HP-bulk lines, respectively

The distribution of the SPC in the RIL and F_2_ population is shown in Table S1 and Fig. 1. In the RIL population, SPC varied continuously, and transgressive segregation was observed. The SPC of high-protein parent ‘Nanxiadou 25’ was consistently higher than that of ‘Tongdou 11’ in two tested locations for 3 years. On average, ‘Nanxiadou 25’ had a 7.18% higher SPC than that of ‘Tongdou 11’. Additionally, the SPC of the RIL population in NC is higher than that in LS. The coefficient of variation (CV) of SPC in RILs ranged from 3.02 to 4.61, and the generalized heritability was as high as 86.68%. The phenotypic data of approximate normal distribution indicated that the mapping population was suitable for QTL analysis. Two-way ANOVA was performed of SPC by SPSS 20.0 software for the RILs population, the genotype (G), environment (E), and genotype by environment interaction (G × E) exhibited significant effects on SPC (*p* < 0.001). For the F_2_ population, the results showed that the frequency of SPC was approximately normally distributed and the tremendous transgressive segregation for SPC was observed. The high-protein parent ‘Nanxiadou 25’ had a 9.33% higher of SPC than the wild type ‘Rongxiandongdou’.

### Genetic map and QTL analysis of SPC

**Fig. 2:**
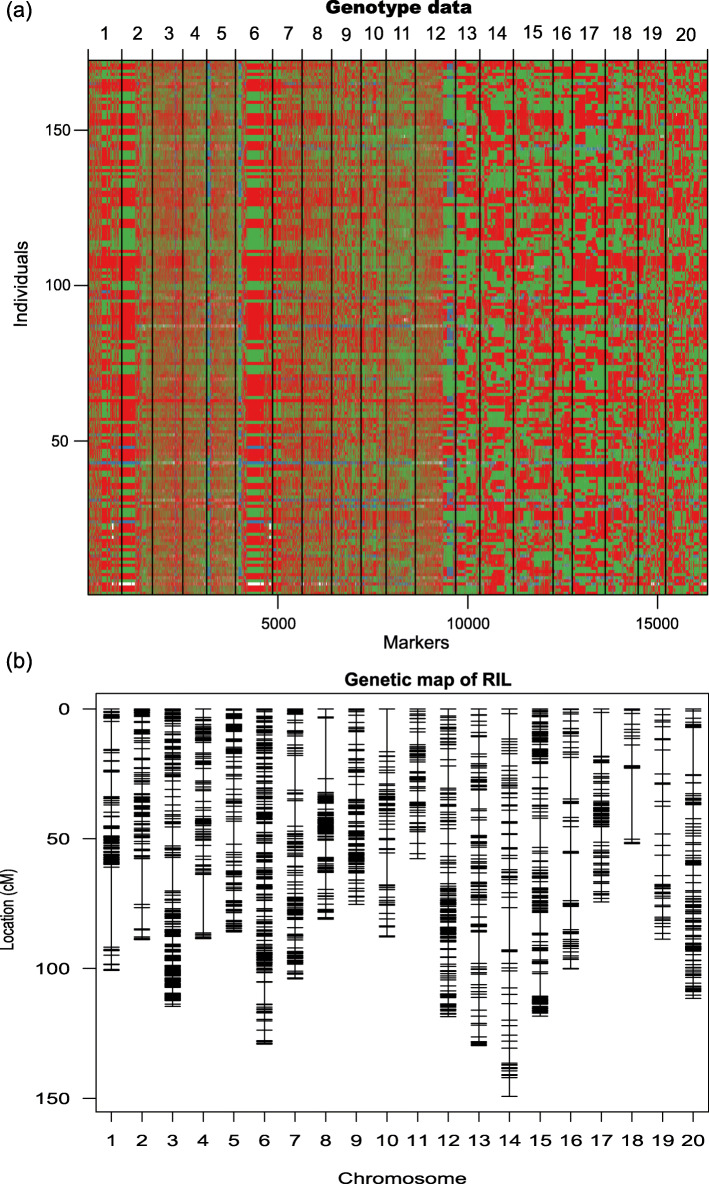
The construction of the SNP-map for the RIL population. (a) Genotype data of individuals, green and red bars represent segments from ‘Nanxiadou 25’ and ‘Tongdou 11’ genotypes, respectively; (b) The distributions of bins on 20 chromosomes

A total of 28,364 SNP markers from the 50 K array showed polymorphisms between the mapping parents ‘Nanxiadou 25’ and ‘Tongdou 11’. SNPs with severe segregation distortion (x^2^ test, *p* < 0.05) were removed through Joinmap 4.0. Among these, 16,546 homologous SNP markers showing the expected segregation 1:1 ratio in the RIL population were used for genetic linkage analysis and linkage map construction using the MPR method (Fig. 2a). The R/qtl software package was used to draw high-density genetic map (Fig. 2b). The final map included 2072 bins covering 1945.09 cM and spanned 20 linkage groups (LGs) with an average distance of 0.94 cM between adjacent bins. There was an average of 104 bins on each LG, ranging from 17 (on Gm18) to 232 (on Gm03) (Table S2 and S3).

**Fig. 3:**
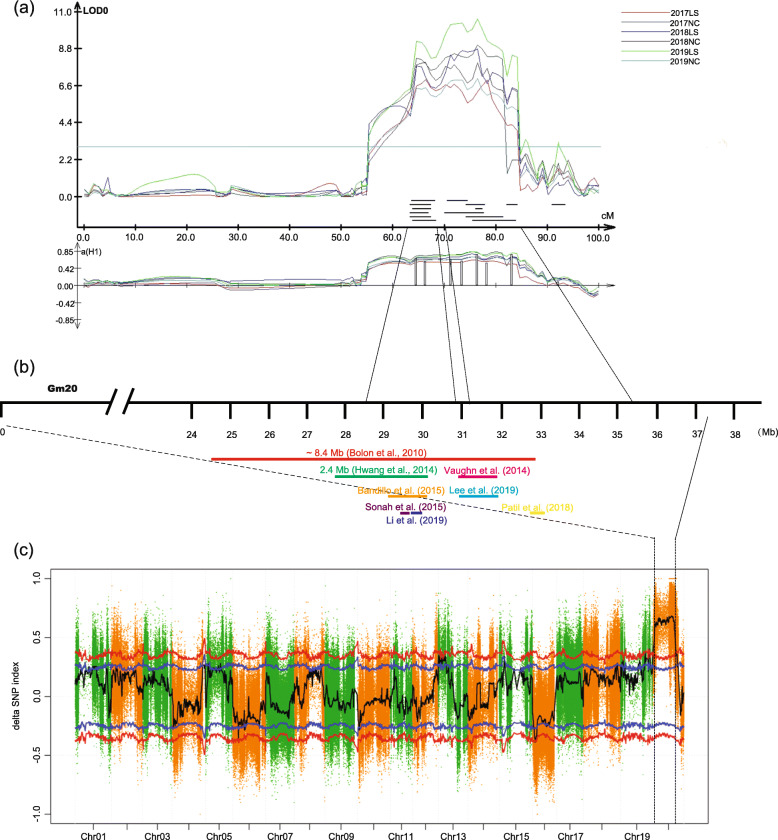
QTL mapping and QTL-seq results. (a), QTL peak map of soybean chromosome 20 from 178 RIL lines across six tested environments. (b), Overlapping relationships among SPC quantitative trait loci (QTLs) in this and previous studies (since 2010). For facilitate comparison, all results are uniformly converted to version glyma 1.0. (c), SNP-index graphs generated from whole-genome resequencing using a QTL-seq analysis. The black lines indicate the average SNP index based on a 1-Mb genomic interval with a 100-kb sliding window. Blue and red lines represent the statistical confidence intervals at significance levels *P* < 0.05 and *P* < 0.01, respectively

Using the genetic map, 50 QTL were mapped on 14 chromosomes except for Chr. 4, 12, 14, 17, 18, and 19 (Fig. S1). These QTL explained 1.21–17.50% of the phenotypic variation for SPC with an average of 63.16% of total phenotypic variation across six tested environments (Table 1). Two closely linked QTL q*SPC*_20–1 and q*SPC*_20–2 explaining 20.13 to 46.88% of the phenotypic variation for SPC across the six tested environments was detected at the linkage group I (Chr 20) (Fig. 3a), followed by the next largest QTL q*SPC*_15 (LOD of 4.06–9.25) (Table 1). For QTL q*SPC*_20–1 and q*SPC*_20–2, it has overlapping confidence intervals with many previous reports, which may be a major QTL (Fig. 3b). The alleles of q*SPC*_20–1 and q*SPC*_20–2 from ‘Nanxiadou 25’ contributed to the increase in SPC, whereas the increase in SPC by QTL q*SPC*_15 on chromosome 15 was contributed by ‘Tongdou 11’. The QTL detected in LS environments are mainly on chromosomes 3, 15, and 20, while the QTL detected in NC environments have a relatively wide range of distribution. Among those QTL, q*SPC*_16 was only detected in all NC environments on chromosome Gm16 with an effect of 4.67 to 4.98% on the phenotypic variation and suggested it is an environmental specific site.

**Table 1:** Principal Characteristics of QTL for SPC in six tested environments

Environment	QTL name	Chromosome	Position (bp)	Physical Region (bp)	LOD	***R***^**2**^	Additive effect	PVE (%)
2017LS	q*SPC*_03	Gm03	15,202,009	14,778,473–16,144,154	3.13	2.61	0.23	71.01
	q*SPC*_06	Gm06	26,103,041	26,048,656–27,047,960	3.53	6.26	0.5	
	q*SPC*_06	Gm06	51,046,599	49,177,878–51,255,865	4.13	4.83	0.35	
	q*SPC*_07	Gm07	40,736,523	40,573,526–40,801,871	5.14	9.25	−0.6	
	q*SPC*_08	Gm08	46,138,625	44,345,029–47,400,674	2.68	4.86	−0.41	
	q*SPC*_15	Gm15	28,480,017	27,758,348–28,637,600	4.49	14.2	−0.71	
	q*SPC*_15	Gm15	31,460,738	30,932,029–32,480,047	7.56	8.87	−0.56	
	q*SPC*_20–1	Gm20	32,752,215	29,941,825–33,575,096	6.97	9.99	0.54	
	q*SPC*_20–2	Gm20	35,324,637	34,001,651–36,397,119	6.94	10.14	0.55	
2017NC	q*SPC*_01	Gm01	31,005,638	30,066,989–31,278,484	2.91	3.28	−0.44	75.93
	q*SPC*_07	Gm07	40,736,523	40,573,526–40,801,871	3.66	5.94	−0.54	
	q*SPC*_09	Gm09	30,262,482	29,336,980–31,646,725	3.71	5.08	0.38	
	q*SPC*_09	Gm09	34,189,648	33,514,884–34,644,806	3.26	4.49	0.31	
	q*SPC*_10	Gm10	15,015,727	13,820,762–17,377,372	3.48	5.8	0.64	
	q*SPC*_10	Gm10	22,505,497	21,845,895–22,873,926	6.77	11.52	1.16	
	q*SPC*_15	Gm15	28,480,017	27,758,348–28,637,600	6.41	11.7	−0.69	
	q*SPC*_16	Gm16	6,616,428	6,570,336–6,706,066	3.24	4.86	−0.45	
	q*SPC*_20–1	Gm20	32,752,215	30,262,326–33,575,096	8.02	11.07	0.59	
	q*SPC*_20–2	Gm20	35,320,625	34,001,651–35,324,637	8	12.19	0.65	
2018LS	q*SPC*_03	Gm03	15,202,009	14,808,506–15,561,956	3.02	1.58	0.16	40.19
	q*SPC*_15	Gm15	28,480,017	27,758,348–28,637,600	4.06	7.71	−0.56	
	q*SPC*_20–1	Gm20	32,752,215	30,215,156–33,722,368	8.26	13.83	0.66	
	q*SPC*_20–2	Gm20	35,320,625	34,001,651–35,324,637	9.04	17.07	0.76	
2018NC	q*SPC*_01	Gm01	31,005,638	30,066,989–31,278,484	2.83	4.09	−0.45	81.08
	q*SPC*_02	Gm02	2,676,772	1,853,600–3,366,836	3.21	1.84	0.19	
	q*SPC*_02	Gm02	7,669,124	6,198,717–8,753,999	3.49	2.47	0.23	
	q*SPC*_03	Gm03	323,364	204,043–561,263	3.49	1.44	−0.37	
	q*SPC*_03	Gm03	15,202,009	14,778,473–15,561,956	3.79	1.38	0.15	
	q*SPC*_05	Gm05	7,963,945	7,788,051–7,989,411	2.9	2.02	0.2	
	q*SPC*_09	Gm09	34,189,648	33,092,853–34,644,806	3.21	3.49	0.26	
	q*SPC*_13	Gm13	885,796	141,952–2,463,480	3.27	3.42	0.29	
	q*SPC*_15	Gm15	28,480,017	27,849,290–28,637,600	3.75	8.15	−0.58	
	q*SPC*_15	Gm15	49,230,170	48,924,129–49,330,218	4.91	1.22	−0.28	
	q*SPC*_16	Gm16	6,616,428	6,570,336–6,706,066	3.13	4.67	−0.45	
	q*SPC*_20	Gm20	32,752,215	30,262,326–33,722,368	9.25	12.54	0.63	
	q*SPC*_20–1	Gm20	35,320,625	34,001,651–35,324,637	8.43	17.5	0.76	
	q*SPC*_20–2	Gm20	35,364,671	35,363,119–36,473,914	10.6	16.85	0.77	
2019LS	q*SPC*_01	Gm01	28,817,299	28,625,066–30,066,989	4.08	7.65	−0.56	69.77
	q*SPC*_11	Gm11	10,518,944	10,183,893–11,819,989	4.25	6.54	0.55	
	q*SPC*_15	Gm15	28,480,017	27,849,290–28,637,600	3.35	8.56	−0.58	
	q*SPC*_15	Gm15	31,460,738	30,932,029–32,085,742	4.77	6.74	−0.5	
	q*SPC*_20	Gm20	32,752,215	30,262,326–33,575,096	7.81	11.83	0.62	
	q*SPC*_20–1	Gm20	35,089,898	33,744,434–34,286,637	8.45	13.51	0.64	
	q*SPC*_20–2	Gm20	35,320,625	34,133,367–36,374,707	8.81	14.94	0.7	
2019NC	q*SPC*_03	Gm03	323,364	0–561,263	3.31	1.41	−0.34	40.98
	q*SPC*_15	Gm15	28,480,017	27,849,290–28,637,600	3.33	7.57	−0.55	
	q*SPC*_15	Gm15	31,460,738	30,932,029–32,600,478	4.18	6.57	−0.51	
	q*SPC*_16	Gm16	6,616,428	6,570,336–6,706,066	3.54	4.98	−0.45	
	q*SPC*_20–1	Gm20	32,752,215	29,558,474–33,575,096	6.5	9.77	0.57	
	q*SPC*_20–2	Gm20	35,324,637	33,744,434–35,514,013	6.96	10.68	0.6

### QTL-seq identified a major SPC QTL on chromosome 20

Based on phenotyping data of SPC among the F_2_ mapping population, two extreme bulks and two-parent bulks were used to construct libraries and subjected to whole-genome sequencing on the Illumina X-ten system. After filtering, the high quality of the sequencing data was obtained with an average Q20 of 94.9% and Q30 of 87.6% (Table S4). An SNP-index was calculated for each identified SNP by computed at a 1 Mb interval using a 1 kb sliding window and was plotted for the HP-bulk (Fig. S2a) and LP-bulk (Fig. S2b). The Δ (SNP-index) calculated and plotted against the genome positions by combining the information of SNP-index in HP-bulk and LP-bulk (Fig. 3c). At the 99% statistical level, a 37.88 Mb on chromosome 20 from 0.01 to 37.89 Mb was significantly correlated with SPC (Fig. 3c, Table S5), named qSPC-I. Compared with the results of linkage analysis, this candidate region overlaps the confidence interval of QTL q*SPC*_20–1 and q*SPC*_20–2. These results indicated that there was a major QTL related to SPC on chromosome 20. Moreover, at the 95% statistical level, the genomic region had a Δ (SNP index) value that was significantiy different from 0 was detected on chromosomes 1, 2, 5, 6, 10, 13, 15, 16, 18, and 19, respectively (Table S6). For qSPC-I, 46,530 SNPs were identified in parental lines and 61 of them had an SNP-index of 1.0 in the HP-bulk indicating that the reads contained genomic fragments derived from ‘Nanxiadou 25’). Of all these SNPs, 778 could result in changes in coding sequences (Table S7).

### Identification of SPC–related candidate genes from the reliable QTL

To explore the candidate genes related to SPC, RNA-seq analysis was performed using RNA extracted from developing seeds of Nanxiadou 25 and Rongxiandongdou at growth stages R5, R6, and R7. Using the criteria of |log2 (fold change)| > 1.5 and a *P*-value ≤0.05, 6440, 6051, and 4795 DEGs showed significantly different expression between Nanxiadou 25 and Rongxiandongdou. Among them, the expression of 86 genes increased significantly in three periods, and that of 555 genes decreased significantly in three periods (Fig. S3). To screen the candidate genes more efficiently, we selected the credible loci from the colocalization interval of the QTL and QTL-seq results. Because the q*SPC*_20–1 and q*SPC*_20–2 were repeatedly detected across six tested environments and overlapped with qSPC-I from QTL-seq, and we analyzed the expression profiles for all genes within the QTL region of q*SPC*_20–1 and q*SPC*_20–2 and removed some non-differentially expressed genes based on the RNA-seq data. A total of 329 candidate DEGs were obtained (Table S8).

**Fig. 4:**
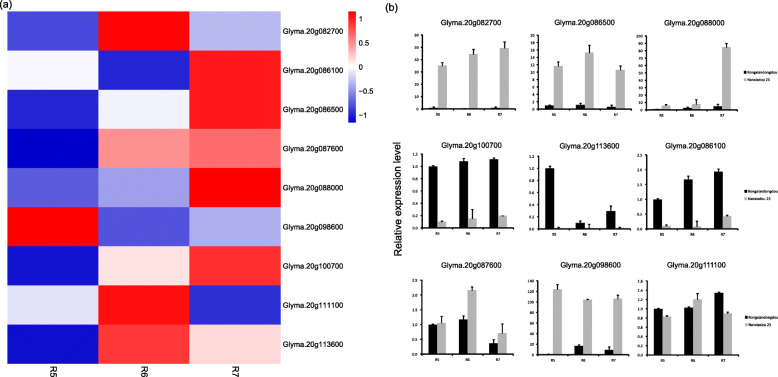
Expression patterns of the nine most promising candidate genes. (a), Heat map of the expression profiles of candidate genes at different developmental phases. (b), Expression profiles of the seven most promising candidate genes via qRT-PCR. Data is the mean of three biological replicates + SE

To narrow down the candidate genes for SPC, based on the gene annotation information of the soybean reference genome, we chose the nine most promising candidate genes, including Nodulin MtN3 family protein (*Glyma.20 g082700*), actin-related protein C2B (*Glyma.20 g086100*), zinc knuckle family protein (*Glyma.20G086500*), Target SNARE coiled-coil domain protein (*Glyma.20 g087600*), S-adenosyl-L-methionine-dependent methyltransferases superfamily protein (*Glyma.20G088000*), tonoplast intrinsic protein (*Glyma.20 g098600*), nuclease (*Glyma.20G100700*), a member of Synaptobrevin-like protein family (*Glyma.20G111100*), and bZIP transcription factor family protein (*Glyma.20 g113600*) (Fig. 4a). We further validated the expression levels of these ten genes by qRT-PCR analysis between the two parents. The qRT-PCR results were similar to those from RNA-seq analysis, suggesting that our RNA-seq results were reliable (Fig. 4b).

### Candidate genes for SPC screening

**Fig. 5:**
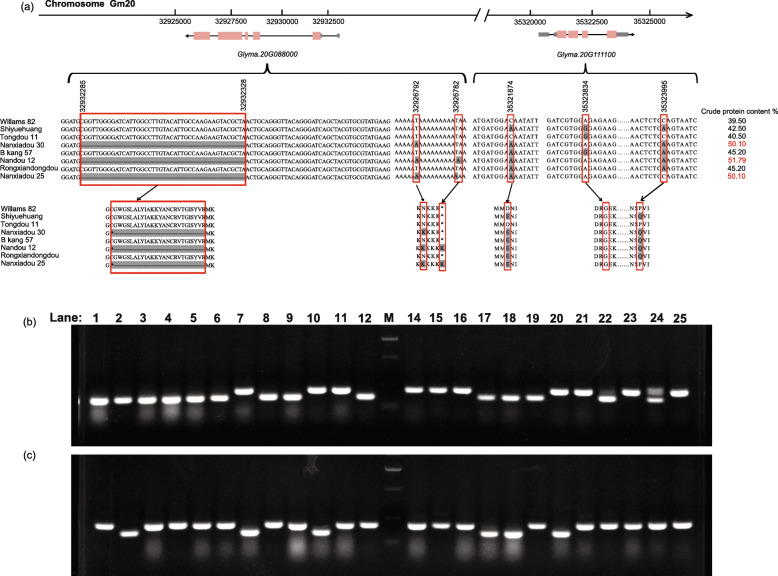
Sequencing analysis and specific SNPs identified in eight different soybean genotypes. (a), Sequencing analysis of two genes in eight different soybean genotypes. (b), Identification of polymorphism for InDel marker in natural population. M: marker. 1–12 lanes and 14–25 lanes correspond to the varieties with large difference in protein content. The detailed information is shown in Table S9. (c), Identification of polymorphism for InDel marker in RILs population. M: marker. 1–12 lanes and 14–25 lanes correspond to the lines with large difference in protein content. The detailed information is shown in Table S9

To further explore the causal gene of SPC, we compared the SNP information of the nine most promising candidate genes between ‘Nanxiadou 25’ and ‘Rongxiandongdou’. After filtering out the meaningless mutation information, we further narrowed down the scope to three candidate genes *Glyma.20G088000*, *Glyma.20G111100* and *Glyma.20 g087600*. To eliminate accidental variation, we compared the sequences of three potential candidate genes in ‘Nanxiadou 25’ and ‘Rongxiandongdou’ and four other soybean genotypes. Because the ‘Nanxiadou 25’ accession was from ‘Rongxiandongdou’, we also evaluated the three potential candidate genes sequence polymorphism from 2 additional accessions (B kang 57 and Nandou 12) from ‘Rongxiandongdou’. For *Glyma.20G088000*, which is annotated as an S-adenosyl-L-methionine-dependent methyltransferases superfamily protein involved in the lipid biosynthetic process, 47 SNPs and 12 InDels were identified among the eight soybean genotypes. Among these SNPs, two SNPs were specific in high protein varieties: the one at 32,926,792 bp was a T–A substitution that caused the amino acid change from Asn to Lys; the other one at 32,926,782 bp only found in ‘Nandou 12’ and ‘Nanxiadou 25’ that caused the terminator codon mutation. Also, there is a 44 bp deletion mutation at sites 32,932,285 and 32,932,328 bp leading to produce a stop codon, and only existing in high protein varieties (Fig. 5a). *Glyma.20G111100*, which is annotated as encodes a member of the synaptobrevin-like protein family, is required for trafficking of storage proteins to the protein storage vacuoles (PSV) and also for PSV organization and biogenesis. A total of 18 SNPs and 2 InDels were identified in *Glyma.20G111100* among the eight soybean genotypes, and two SNP variations led to amino acid changes (Fig. 5a).

*Glyma.20 g087600* is annotated as a target SNARE coiled-coil domain protein involved in ER to Golgi vesicle-mediated transport. Although there is no mutation in the gene sequence, its great potential was located near the peak SNP (32,752,215 bp) which was repeatedly detected by multiple environments, and Lee et al. (2019) reported that this SNP related to soybean seeds protein and oil content [5]. In order to evaluate the potential of this SNP, we further analyzed the natural variation of this locus using protein content data obtained from the GRIN database and genotype data obtained from SoyBase database. Interestingly, we found that the allele “C” frequency was 0.61 in the population with protein content greater than 50%. However, the proportion decreased to 0.002 in the population with protein content less than 42%, and only seven varieties from Japan had the allele “C” in this site (Fig. S4a-b).

In addition, the qSPC_16 only from the NC environment is another focus of our attention. In this QTL region, *Glyma.16G066600* which is annotated as an alpha-vacuolar processing enzyme involved in vacuolar protein processing has brought our attention. In *Glyma.16G066600*, between the eight identified SNPs, only one SNP at 6,606,140 bp was a G–T, which led to the amino acid change from Ser to Ile and was not specific to high protein varieties (Fig. S4c).

### Validation of the association between variation and phenotype in the natural and RILs populations

We selected *Glyma.20 g088000* for validating the association between the variation and phenotype in the natural and RILs populations. A total of 96 lines randomly selected from natural and RIL populations were used to amplify the DNA fragments harboring the significant SPC-related InDel that was located within the 1st exon of *Glyma.20 g088000*. PCR amplification showed that there was an about 160 bp electrophoresis band in high protein lines, and about 204 bp in low protein lines (Fig. 5b-c, Table S9). A t-test was then conducted on the phenotype of SPC between the two groups in each environment of RILs and natural populations. As a result, the phenotype of the lines containing the deletion-allele was significantly (*P* < 0.05) larger than that of the lines containing the normal-allele in each of the environments (Table S10).

## Discussion

SPC, as a typical quantitative trait, is controlled by multiple minor effects genes [3]. Since the availability of genetic linkage map in soybean, a large number of QTL have been identified for soybean SPC using linkage mapping in biparental segregation populations and association mapping in natural populations in the recent thirty years [33]. According to incomplete statistics, these QTL were widely distributed in different regions of all 20 chromosomes. Among these chromosomes, chromosome 20 had remarkable attention due to its high additive effect (12–55% phenotypic variation) and stability [34]. The genomic region (24.5 to 32.9 Mb) on chromosome 20 is a particularly attractive major common QTL for SPC [4, 10, 17]. Subsequently, Hwang et al.(2014) further narrowed this region to a 2.4-Mb region located between 27.6 and 30.0 Mb [4]. Interestingly, Vaughn et al. (2014) [35] reported a QTL approximately 1 Mb downstream of the region that Hwang et al. (2014) identified [4], and Lee et al. (2019) further identified an 839 kb region within this 1 Mb genomic region by GWAS [5]. However, Patil et al. (2018) reported a QTL highly associated with SPC using a high-resolution bin map located in 33,975,596–34,027,051 bp [34], and Li et al. (2019) reported the other one genomic region (30995685–31,177,423 bp) highly associated with SPC using multi-locus genome-wide association studies [33]. In addition, Chung et al. (2013) identified the 30.5–32.3 Mb regions on chromosome 20 formed by long-term domestication and selection [36]. Vaughn et al. (2014) further confirmed that this region may be related to domestication and selection based on the result of the Tajima’s D values [35]. It is generally accepted that there is a genetic locus controlling the protein content of soybean on chromosome 20, but there are different conclusions about the exact location of the genetic locus. In our study, at the 99% statistical level, one genomic region on chromosome 20 from 0.01 to 37.89 Mb had a Δ (SNP-index) value that was significantly associated with SPC. This result further proves that there is a stable and reliable genetic locus of SPC on chromosome 20. Interestingly, we detected two stable QTL on chromosome 20 by linkage mapping, and the two QTL were close to each other and overlapped with the previously reported QTL regions (Table 1 and Fig. 3a). Combined with the differences of previous results, we speculated that there might be two or more genetic loci controlling protein content on chromosome 20.

From the detected QTL, many candidate genes were identified for SPC. Bolon et al. (2010) found that 13 genes displaying significant seed transcript accumulation differences between NILs were identified that mapped to the 8.4 Mbp QTL region on LG I by AffymetrixÂ® Soy GeneChip and high-throughput IlluminaÂ® whole transcriptome sequencing platforms [17]. Bandillo et al. (2017) further narrowed this region and this region now encompassed only three (*Glyma20g21030*, *Glyma20g21040*, and *Glyma20g21080*) of the original 12 potential candidate genes [10]. Moreover, 13 genes were revealed in the confidence interval QTL on Chr.20 that was flanked by bin_20_33,975,596 and bin_20_34,027,051 markers based on high-density linkage mapping [34], and *Glyma20g21693* and *Glyma20g21726* were considered the important genes within the other one region (Gm20_30,995,685–31,177,423 bp) [33]. To identify candidate genes within the major QTL regions on Chr.20, we performed RNA-seq in Nanxiadou 25 and Rongxiandongdou, revealing some key genes that might be involved in seed storage albumins biosynthesis in soybean. Compared with the high protein genotype Nanxiadou 25, the low protein parent Rongxiandongdou had more upregulated genes and fewer downregulated genes, showing that the high protein genotype to the accumulation of storage protein was mainly based on the downregulated genes, while the low protein genotype to the accumulation of storage protein was based on the positive of genes. Our experiments identified nine most promising DEGs with the significant differences in major QTL regions by RNA-seq analysis, and nine DEGs were further screened by qRT-PCR and cloning and sequencing analysis. Finally, three candidate genes (*Glyma.20G088000*, *Glyma.20G111100,* and *Glyma.20 g087600*) were considered the important genes within this region. Many reports have shown that there is a negative correlation between SPC and oil content and seed yield [2, 3], and there is obvious co-location on chromosome 20 [5, 10, 33, 34]. Although we have not analyzed the seed oil content in this study, we believe that *Glyma.20 g088000* having a huge difference sequence between high and low protein varieties and having high expression in Nanxiadou 25 may be related to the high protein formation of Nanxiadou 25. The peak SNP (32,752,125 bp) was repeatedly detected in multiple environments, and Lee et al. [5] reported that this SNP related to seeds protein and oil content. According to the germplasm statistics of the GRIN database, the rare allele C of this SNP locus hardly appeared in the low protein varieties. Therefore, as the nearest gene to SNP, we surmised that *Glyma.20 g087600* has great potential for SPC. However, the relationship between SNP and the gene remains to be elucidated.

Except for chromosome 20, QTL on chromosome 15 was the most reported. We also detected several QTL on 13 other chromosomes including chromosome 15. As a typical quantitative trait, we are more interested in QTL only detected in the NC environment than those repeatedly detected in multiple environments but with low *R*^2^. The q*SPC*_16 only found in the NC environment that had a huge latitude difference with LS, indicating that q*SPC*_16 was an environmental specific site. Based on QTL-seq, we identified a qSPC-J-1 in the same region, which further indicated that q*SPC*_16 was stable in the NC environment. *Glyma.16G066600*, which was considered the important genes within this region, is annotated as a vacuolar processing enzyme (also known as aspartic protease, APE) involved in seed storage protein processing under standard growth conditions. In conclusion, we identified four potential candidate genes by combining QTL mapping, QTL-seq, RNA-seq, and cloning sequencing. However, we remain cautious about the above results. These genes are involved in different biological functions, suggesting that SPC was a complex trait that involves a series of biochemical pathway-related genes. Therefore, more in-depth studies are needed to validate the functions of candidate genes, and insight into the genetic and molecular control mechanisms involved in the deposition of SPC in the developing seed to guide crop improvement.

## Conclusions

In this study, we performed quantitative trait loci (QTL) mapping, QTL-seq, and RNA sequencing (RNA-seq) to reveal the genes controlling protein content in the soybean by using the high protein content variety Nanxiadou 25 and the low protein content variety Tongdou 11. A total of 50 QTL for SPC distributed on 14 chromosomes were identified by QTL mapping using 178 recombinant inbred lines (RILs). Among these QTL, the major QTL on chromosome 20 were repeatedly detected across six tested environments, corresponding to the location of the major QTL detected using whole-genome sequencing-based QTL-seq. 329 candidate DEGs were obtained within the QTL region of q*SPC*_20–1 and q*SPC*_20–2 via gene expression profile analysis. Nine of which were associated with SPC, potentially representing candidate genes. Clone sequencing results showed that different single nucleotide polymorphisms (SNPs) and indels between high and low protein genotypes in *Glyma.20G088000* and *Glyma.16G066600* may be the cause of changes in this trait.

## Methods

### Plant materials and phenotypic evaluation

A population of 178 F_7_ RILs was derived by single seed descent from F_2_ offspring of a cross between cultivar Nanxiadou 25 (high SPC, 50.1%) and cultivar Tongdou 11 (low SPC, 40.5%). These RILs and their parents were grown in six environments, summer of 2017, 2018, and 2019 in the Nanchong academy of agricultural sciences, Sichuan, China (NC; 30.87°N, 106.04°E), and winter of 2017, 2018, and 2019 in the Lingshui off-season breeding base, Hainan, China (LS; 18.53°N, 110.01°E). The lines were arranged in a randomized complete block design with three replicates in a single row plot with a 1.0-m row length and 0.5-m row spacing. A total of 2097 F_2_ plants, which derived from a cross of cultivar Nanxiadou 25 and landraces Rongxiandongdou (wild type of Nanxiadou 25, low SPC), were grown in Nanchong academy of agricultural sciences in 2019. B kang 57 (a natural mutant of Rongxiandongdou, low SPC), Nandou 12 (Mutagenic progeny of B kang 57, high SPC), Nanxiadou 30 (High protein cultivar), Shiyuehuang (a soybean landraces with low protein content), and Williams 82 came from the germplasm resources preserved in Nanchong academy of agricultural sciences.

A NIR System 6500 with WinISI II software (FOSS GmbH, Denmark) was used to measure SPC with approximately 20 g whole seeds of a 13% moisture basis. The wavelength range covered was from 950 to 1650 nm. The mean value of three scans of each sample was used in data analysis. Basic statistical analysis of the phenotype data was performed using SPSS software.

### Map construction and QTL analysis

Qualified libraries were paired-end sequenced on the Illumina Hiseq Xten platform to obtain high-quality SNPs widely distributed throughout the genome. Genetic linkage analysis was performed using the software packages MSTmap [37] and Joinmap v. 4.0 [38]. Firstly, polymorphic SNPs were grouped by MSTmap at LOD 5.0, and then the minimum spanning tree of a graph for each linkage group is found to determine the markers order according to the pairwise recombination frequency. The markers order and distance in each linkage group were recalculated and confirmed by Joinmap 4.0, applying a minimum LOD score of 3.0.

QTL analysis was performed by the QTL Cartographer software version WinQTLCart 2.5 with the Composite Interval Mapping (CIM) method [39]. In CIM analysis, the walking speed of 1-cM was selected, and the regression parameters were 1000 times, with a significance level of 0.01. There may be a QTL in this interval when LOD ≥ 3.0.

### QTL-seq

For QTL-seq, two DNA bulks, higher SPC bulk (HP-bulk) and lower SPC bulk (LP-bulk), were constructed, respectively, by mixing an equal amount of DNA from 30 higher SPC (SPC = 49.72–52.07%) and 30 lower SPC (SPC = 40.34–42.06%) F_2_ individuals from the 2019 experiment. Two parent bulks were constructed by 5 random single plants, respectively. Four paired-end sequencing libraries were constructed with about 5 mg DNA from two bulks and two parental bulks and sequenced on the Illumina sequencing platform by Genedenovo Biotechnology Co., Ltd. (Guangzhou, China). After removing adapter and low quality reads, high-quality sequences were aligned to the Williams 82 reference genome using BWA software [40]. Genome Analysis Toolkit (GATK) was used to call SNPs and small indels across parental lines and bulks [41].

SNP-index was calculated for all the SNP positions by sliding window analysis. Removing spurious SNPs, that SNP-index of < 0.3 or > 0.7 and read depth less than 7, called due to sequencing and/or alignment errors. The SNP index of two bulks was subtracted to get Δ (SNP-index), and QTL was identified in these positive or negative peak regions with 95% confidence interval in 10,000 bootstrap replicates. Then, selected SNPs and InDels in the peak regions to annotate and screened potential functional variants.

### RNA-seq and expression analysis of candidate genes

RNA-seq and real-time quantitative PCR (RT-qPCR) was used to investigate the expression pattern of the candidate genes in Co-localization of QTL-seq and linkage mapping. Each flower marked at the early flowering stage and randomly collected five pods from each of the three different plants of two parents at growth stages R5, R6, and R7, respectively. RNA extracted from different plant pods at each developmental stage was mixed equally to construct the library. Removing low-quality reads, all cleaned reads were mapped to the Williams82.a2 using TopHat version 2.0 [42]. The differentially expressed genes (DEGs) were dug by the EBSeq package of R with a FDR (false discovery rate) < 0.05 and |log2 (fold change)| > 1.5.

A total of nine candidate genes were selected for qPCR analysis to verify the reliability of RNA-seq results. According to the manufacturer’s instructions, a 1-μg RNA sample was used for first-strand cDNA synthesis using iScript^Tm^ cDNA Synthesis Kit (Bio-Rad, Hercules, CA, USA), and the expression levels of nine candidate genes were measured using real-time PCR performed on a CFX96 Real-time System (Bio-Rad). Three technical replications were performed per sample. The relative expression level of each gene was calculated based on the 2^–ΔΔCt^ method [43] using *Actin11* as an internal control. The primers for qRT-PCR were designed by Primer Premier 5.0 and listed in Table S11.

### Sequence analysis of candidate genes

To clarify the variation of the candidate genes from the overlap region of QTL mapping and QTL-seq in the two parents, the gene sequence of *Glyma.20G111100* and *Glyma.20 g087600* from B kang 57, Nandou 12, Shiyuehuang, and Williams 82 were sequenced and analyzed. The candidate gene was subjected to PCR by using TransStart® FastPfu DNA Polymerase, and sent to TSINGKE Biological Technology Co., Ltd. (Chengdu, China) for sequencing after agarose gel electrophoresis. DNA homology alignment used Invitrogen Vector NTI 11.5.1 software.

## Supplementary Information

**Additional file 1: Table S1.** Descriptive statistics, broad sense heritability and F-value from ANOVA for SPC in the soybean RIL and F_2_ population

**Additional file 2: Table S2.** Distribution of SNPs mapped on soybean chromosomes/linkage groups, **Table S3.** The information of high-density SNP map, **Table S4**. Summary of Illumina sequencing data, **Table S6.** QTL mapping of soybean SPC by bulked segregant analysis (BSA), **Table S7.** The information of SNP in coding sequences of gene, **Table S8.** The RNA-seq data of genes within the QTL region of qSPC_20-1 and qSPC_20-2, **Table S9.** Seed protein content of natural population and RILs population, **Table S10.** *P* values of t-test for the SPC-related InDel in different environments, **Table S11.** Primer information of candidate genes for RT-qPCR

**Additional file 3: Table S5.** Detail of SNP-index and annotation for each SNP in the qSPC-I region

**Additional file 4: Figure S1.** Location of quantitative trait loci (QTL) related to protein contents. For simplicity, only show the markers in the QTL confidence intervals, along with the terminal two markers at each end of the QTL-containing chromosomes

**Additional file 5: Figure S2.** Single nucleotide polymorphism (SNP)-index plots of HP-bulk (High protein bulk) and LP-bulk (Low protein bulk). (a), represent the single nucleotide polymorphism (SNP)-index plots of HP-pool. (b), represent the single nucleotide polymorphism (SNP)-index plots of LP-pool

**Additional file 6: Figure S3.** Venn diagram analysis for RNA-seq data showing differentially expressed genes (Nanxiadou 25/Rongxiandongdou) at R5, R6, and R7 stages. (a), venn diagram representing the number of up-regulation DEGs. (b), venn diagram representing the number of down-regulation DEGs

**Additional file 7: Fig. S4.** Allele classification of SNP in cultivated varieties and multiple sequence alignment depicting the amino acid sequence difference of *Glyma.16G066600*. (a), Represents the genotype distribution of population with SPC less than 42%; (b), Represents the genotype distribution of population with SPC greater than 50%. (c), Represents the multiple sequence alignment depicting the amino acid sequence difference of *Glyma.16G066600*

## Data Availability

The datasets generated and analysed during the current study are available in the NCBI SRA database (BioProject ID: PRJNA752801 and PRJNA752694). Other data generated during this study are included in this article and its additional files. The sequence data of genes in this article can be obtained in Phytozome 13 (https://phytozome-next.jgi.doe.gov/) under the following accession numbers: *Glyma.20G088000*, *Glyma.20G111100*, *Glyma.20 g087600*, and *Glyma.16G066600*. All experimental materials are available on request.

## Abbreviations

SPC: Seed protein content
QTL: Quantitative trait loci
RILs: Recombinant inbred lines
MAS: Marker-assisted selection
USDA: United States Department of Agriculture
BSA: Bulked segregant analysis
GWAS: Genome-wide association studies
WGRS: Whole genome resequencing
NIRS: Near-infrared reflectance spectroscopy
NC: Nanchong
LS: Lingshui
CIM: Composite Interval Mapping
HP-bulk: Higher protein content bulk
LP-bulk: Lower protein content bulk
GATK: Genome Analysis Toolkit
SNP: Single nucleotide polymorphism
RT-qPCR: Real time quantitative PCR
DEGs: Differentially expressed genes
FDR: False discovery rate
FPKM: Per million fragments mapped
LGs: Linkage groups
