# Supplementary material for: Genetic mapping high protein content QTL from soybean ‘Nanxiadou 25’ and candidate gene analysis
Source: BMC Plant Biol. 2021 Aug 20;21:388. doi: 10.1186/s12870-021-03176-2 (PMC8377855; doi:10.1186/s12870-021-03176-2)
Supplement: Supplementary file 1 — Additional file 1: Table S1. Descriptive statistics, broad sense heritability and F-value from ANOVA for SPC in the soybean RIL and F2 population [file 12870_2021_3176_MOESM1_ESM.docx]

**Table S1.** Descriptive statistics, broad sense heritability and F-value from ANOVA for seed protein content in the soybean RIL and F_2_ population.

| Population | Environment ^a^ | Parents | | Range (%) | Mean+SD | Variance | CV ^c^ (%) | Skewness | Kurtosis | F Value of Variance Analysis | | | *H*^2^ ^d^ |
| --- | --- | --- | --- | --- | --- | --- | --- | --- | --- | --- | --- | --- | --- |
|  |  | Tongdou 11/Rongxiandongdou ^b^ | Nanxiadou 25 |  |  |  |  |  |  | Genotype (G) | Environment (E) | G*E |  |
| RILs | 17LS | 41.78 | 47.63 | 40.19-48.85 | 44.98+0.13 | 3.02 | 3.87 | -0.09 | -0.25 | 1.12^*** e^ | 1.01^***^ | 0.03^***^ | 86.68% |
|  | 18LS | 40.71 | 47.55 | 38.73-48.94 | 44.67+0.15 | 3.76 | 4.34 | -0.17 | 0.08 |  |  |  |  |
|  | 19LS | 42.47 | 48.93 | 41.20-50.11 | 46.16+0.15 | 3.54 | 4.07 | -0.09 | -0.32 |  |  |  |  |
|  | 17NC | 43.66 | 51.97 | 42.02-53.14 | 48.63+0.16 | 4.19 | 4.22 | -0.35 | 0.45 |  |  |  |  |
|  | 18NC | 42.42 | 50.88 | 41.87-53.48 | 49.16+0.16 | 4.61 | 4.37 | -0.51 | 0.66 |  |  |  |  |
|  | 19NC | 43.78 | 50.94 | 43.20-52.44 | 48.21+0.15 | 3.66 | 3.96 | 0.03 | -0.32 |  |  |  |  |
| F_2_ | 19NC | 41.71 | 51.04 | 40.14-52.07 | 45.39+0.06 | 2.87 | 3.72 | 0.45 | 1.09 |  |  |  |  |

^a^ 17LS, 18LS, 19LS, 17NC, 18NC, 19NC—different environments of Lingshui, Nanchong in 2017, 2018, and 2019. ^b^ Tongdou 11 in RILs population, Rongxiandongdou in F_2_ population. ^c^ CV—coeffcient of variation. ^d^ *H*^2^—broad-sense heritability. e ^**^ p < 0.01; ^***^ p < 0.001.
