# Supplementary figures and images for: Genetic mapping high protein content QTL from soybean ‘Nanxiadou 25’ and candidate gene analysis
Source: BMC Plant Biol. 2021 Aug 20;21:388. doi: 10.1186/s12870-021-03176-2 (PMC8377855; doi:10.1186/s12870-021-03176-2)

a

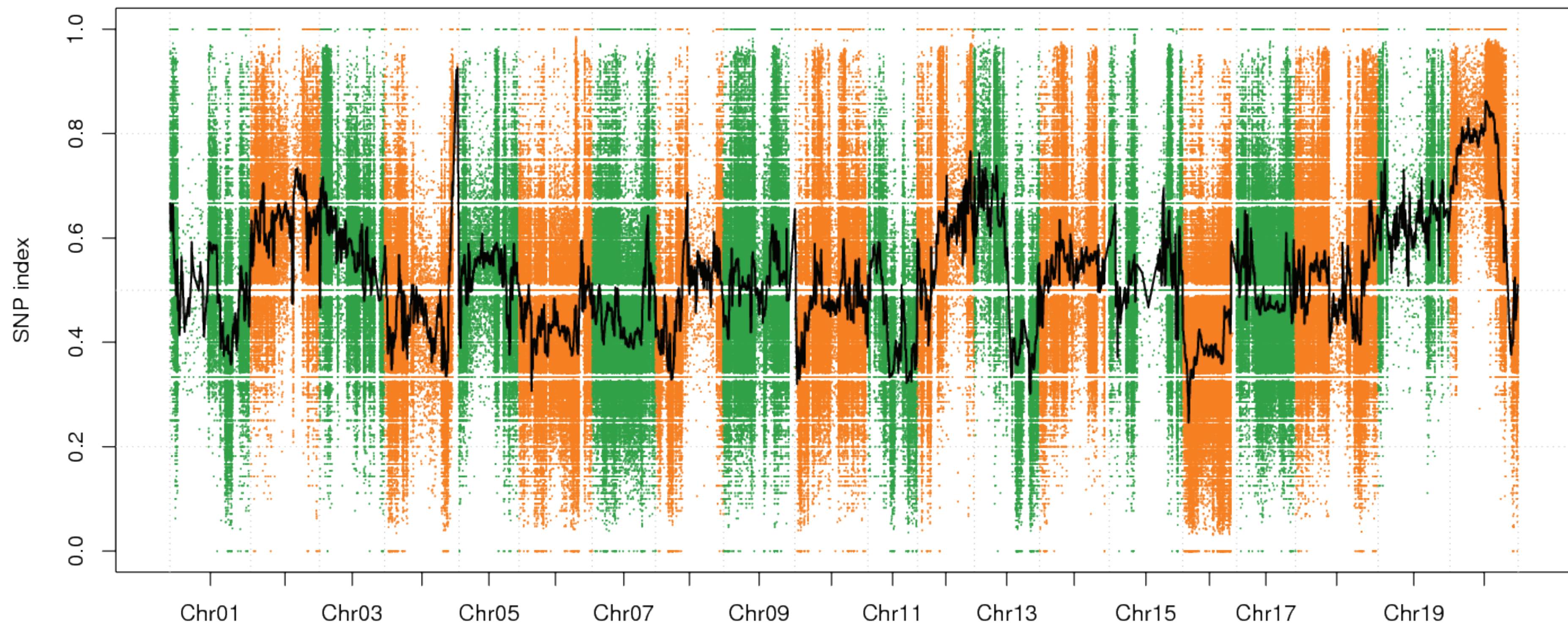

b

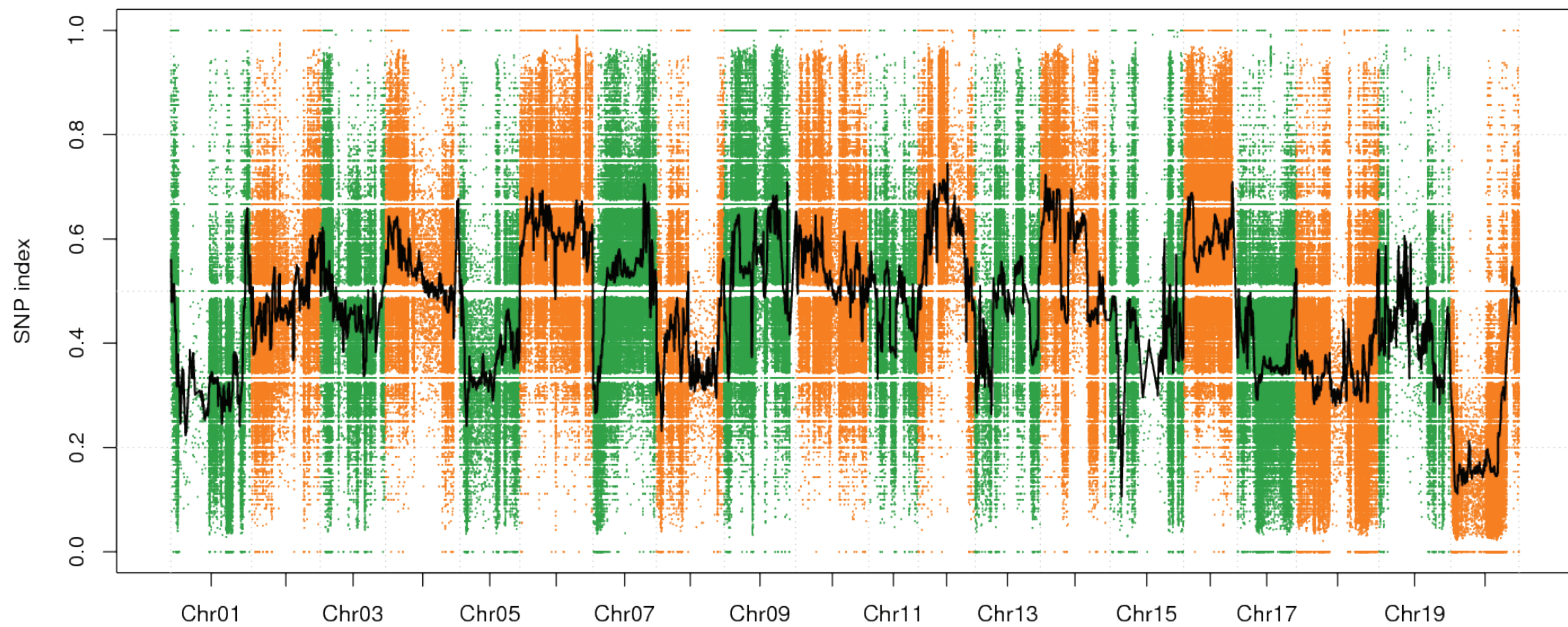

Supplement: Supplementary file 5 — Additional file 5: Figure S2. Single nucleotide polymorphism (SNP)-index plots of HP-bulk (High protein bulk) and LP-bulk (Low protein bulk). (a), represent the single nucleotide polymorphism (SNP)-index plots of HP-pool. (b), represent the single nucleotide polymorphism (SNP)-index plots of LP-pool [file 12870_2021_3176_MOESM5_ESM.pdf]

a

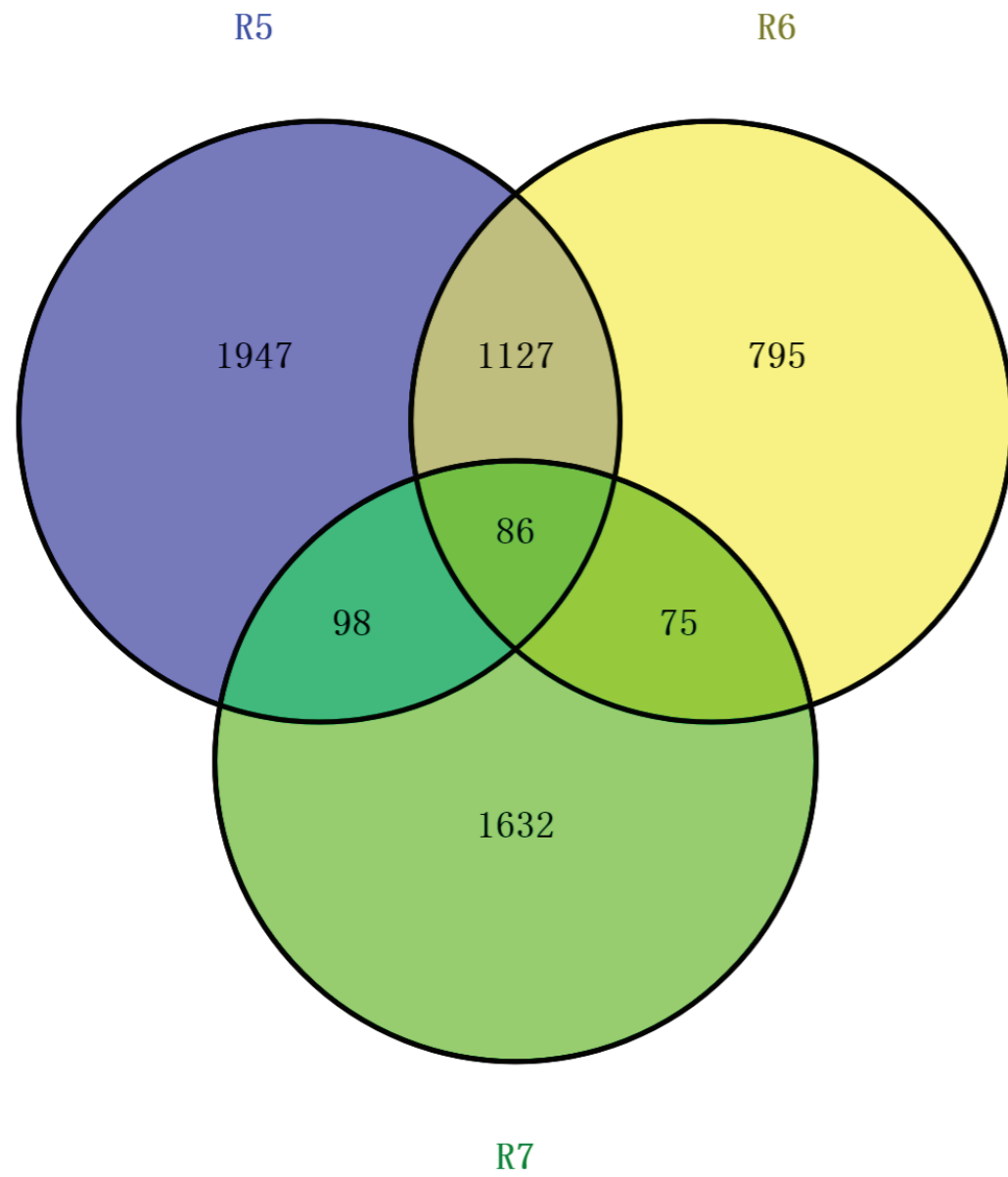

b

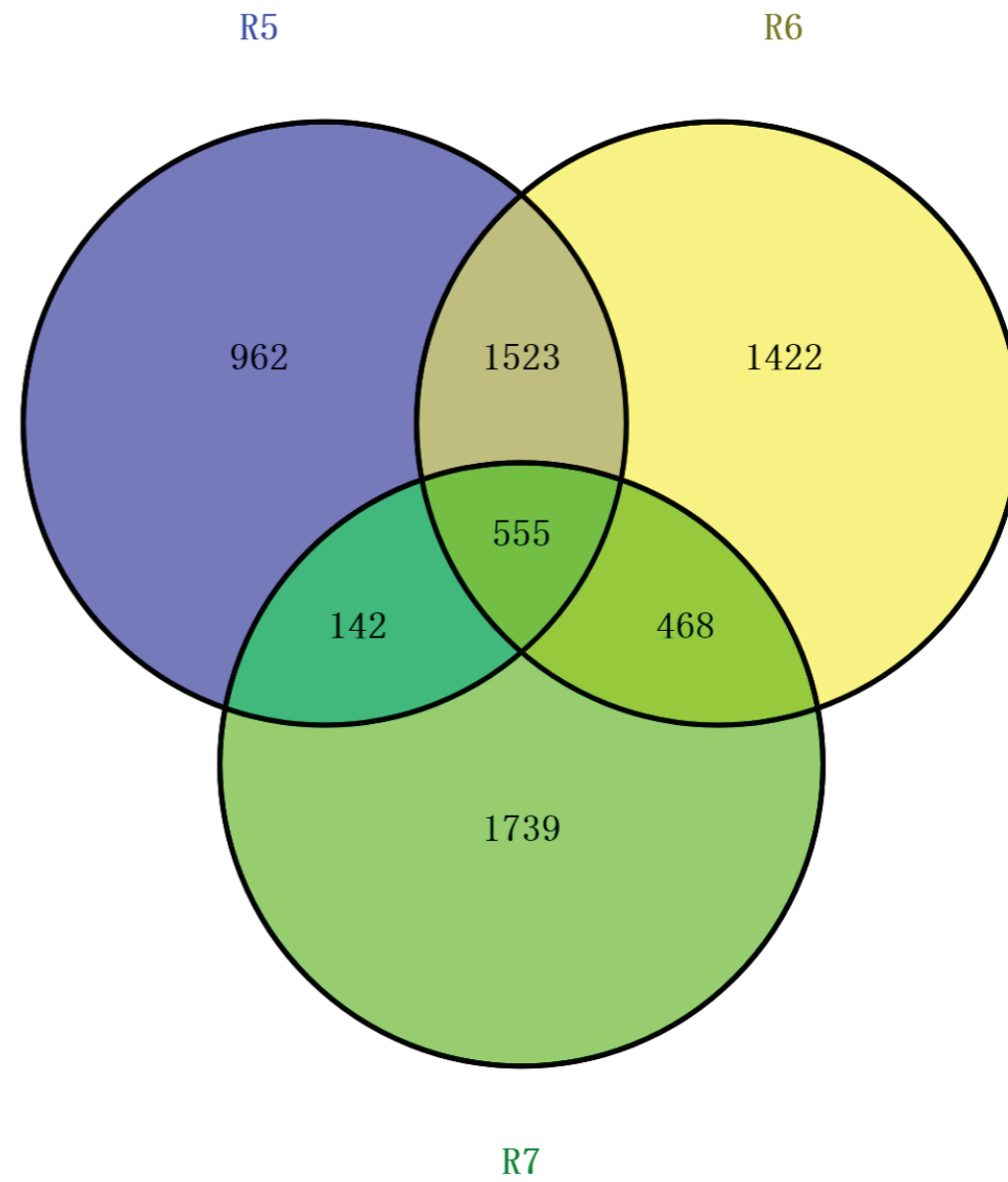

Supplement: Supplementary file 6 — Additional file 6: Figure S3. Venn diagram analysis for RNA-seq data showing differentially expressed genes (Nanxiadou 25/Rongxiandongdou) at R5, R6, and R7 stages. (a), venn diagram representing the number of up-regulation DEGs. (b), venn diagram representing the number of down-regulation DEGs [file 12870_2021_3176_MOESM6_ESM.pdf]
