# Supplementary material for: Genetic mapping high protein content QTL from soybean ‘Nanxiadou 25’ and candidate gene analysis
Source: BMC Plant Biol. 2021 Aug 20;21:388. doi: 10.1186/s12870-021-03176-2 (PMC8377855; doi:10.1186/s12870-021-03176-2)
Supplement: Supplementary file 7 — Additional file 7: Fig. S4. Allele classification of SNP in cultivated varieties and multiple sequence alignment depicting the amino acid sequence difference of Glyma.16G066600. (a), Represents the genotype distribution of population with SPC less than 42%; (b), Represents the genotype distribution of population with SPC greater than 50%. (c), Represents the multiple sequence alignment depicting the amino acid sequence difference of Glyma.16G066600 [file 12870_2021_3176_MOESM7_ESM.pdf]

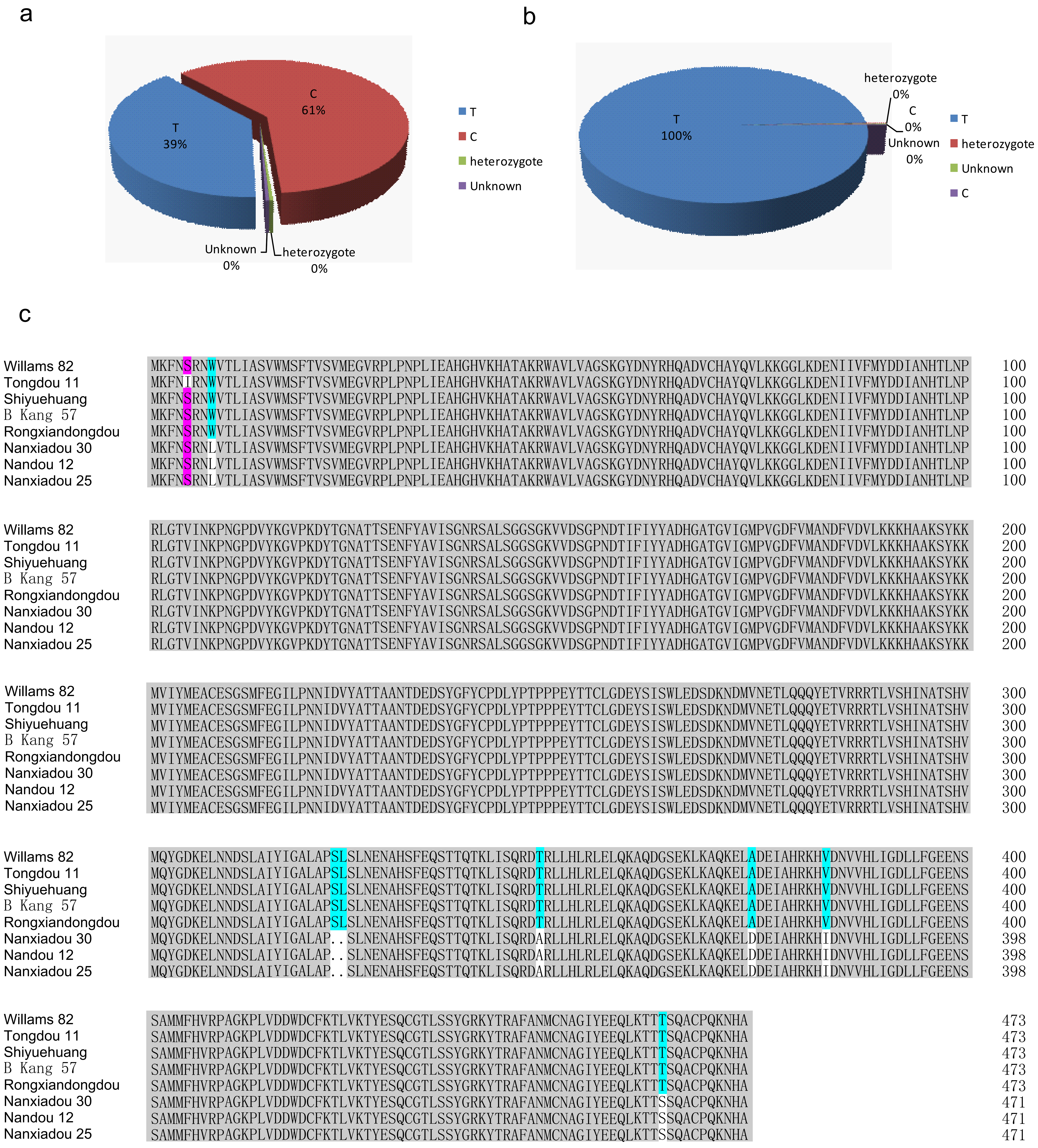
a
T
39%
C
61%
Unknown
0%
heterozygote
0%
T
C
heterozygote
Unknown
b
T
100%
heterozygote
0%
C
0%
Unknown
0%
T
heterozygote
Unknown
C
c
Willams 82 MKFNSRNWVTLIASVWMSFTVSVMEGVRPLPNPLIEAHGHVKHATAKRWAVLVAGSKGYDNYRHQADVCHAYQVLKKGGLKDENIIVFMYDDIANHTLNP 100
Tongdou 11 MKFNIRNWVTLIASVWMSFTVSVMEGVRPLPNPLIEAHGHVKHATAKRWAVLVAGSKGYDNYRHQADVCHAYQVLKKGGLKDENIIVFMYDDIANHTLNP 100
Shiyuehuang MKFNSRNWVTLIASVWMSFTVSVMEGVRPLPNPLIEAHGHVKHATAKRWAVLVAGSKGYDNYRHQADVCHAYQVLKKGGLKDENIIVFMYDDIANHTLNP 100
B Kang 57 MKFNSRNWVTLIASVWMSFTVSVMEGVRPLPNPLIEAHGHVKHATAKRWAVLVAGSKGYDNYRHQADVCHAYQVLKKGGLKDENIIVFMYDDIANHTLNP 100
Rongxiandongdou MKFNSRNWVTLIASVWMSFTVSVMEGVRPLPNPLIEAHGHVKHATAKRWAVLVAGSKGYDNYRHQADVCHAYQVLKKGGLKDENIIVFMYDDIANHTLNP 100
Nanxiadou 30 MKFNSRNLVTLIASVWMSFTVSVMEGVRPLPNPLIEAHGHVKHATAKRWAVLVAGSKGYDNYRHQADVCHAYQVLKKGGLKDENIIVFMYDDIANHTLNP 100
Nandou 12 MKFNSRNLVTLIASVWMSFTVSVMEGVRPLPNPLIEAHGHVKHATAKRWAVLVAGSKGYDNYRHQADVCHAYQVLKKGGLKDENIIVFMYDDIANHTLNP 100
Nanxiadou 25 MKFNSRNLVTLIASVWMSFTVSVMEGVRPLPNPLIEAHGHVKHATAKRWAVLVAGSKGYDNYRHQADVCHAYQVLKKGGLKDENIIVFMYDDIANHTLNP 100
Willams 82 RLGTVINKPNGPDVYKGVPKDYTGNATTSENFYAVISGNRSALSGGSGKVVDSGPNDTIFIYYADHGATGVIGMPVGDFVMANDFVDVLKKKHAAKSYKK 200
Tongdou 11 RLGTVINKPNGPDVYKGVPKDYTGNATTSENFYAVISGNRSALSGGSGKVVDSGPNDTIFIYYADHGATGVIGMPVGDFVMANDFVDVLKKKHAAKSYKK 200
Shiyuehuang RLGTVINKPNGPDVYKGVPKDYTGNATTSENFYAVISGNRSALSGGSGKVVDSGPNDTIFIYYADHGATGVIGMPVGDFVMANDFVDVLKKKHAAKSYKK 200
B Kang 57 RLGTVINKPNGPDVYKGVPKDYTGNATTSENFYAVISGNRSALSGGSGKVVDSGPNDTIFIYYADHGATGVIGMPVGDFVMANDFVDVLKKKHAAKSYKK 200
Rongxiandongdou RLGTVINKPNGPDVYKGVPKDYTGNATTSENFYAVISGNRSALSGGSGKVVDSGPNDTIFIYYADHGATGVIGMPVGDFVMANDFVDVLKKKHAAKSYKK 200
Nanxiadou 30 RLGTVINKPNGPDVYKGVPKDYTGNATTSENFYAVISGNRSALSGGSGKVVDSGPNDTIFIYYADHGATGVIGMPVGDFVMANDFVDVLKKKHAAKSYKK 200
Nandou 12 RLGTVINKPNGPDVYKGVPKDYTGNATTSENFYAVISGNRSALSGGSGKVVDSGPNDTIFIYYADHGATGVIGMPVGDFVMANDFVDVLKKKHAAKSYKK 200
Nanxiadou 25 RLGTVINKPNGPDVYKGVPKDYTGNATTSENFYAVISGNRSALSGGSGKVVDSGPNDTIFIYYADHGATGVIGMPVGDFVMANDFVDVLKKKHAAKSYKK 200
Willams 82 MVIYMEACESGSMFEGILPNNIDVYATTAANTDEDSYGFYCPDLYPTPPPEYTTCLGDEYSISWLEDSDKNDMVNETLQQQYETVRRRTLVSHINATSHV 300
Tongdou 11 MVIYMEACESGSMFEGILPNNIDVYATTAANTDEDSYGFYCPDLYPTPPPEYTTCLGDEYSISWLEDSDKNDMVNETLQQQYETVRRRTLVSHINATSHV 300
Shiyuehuang MVIYMEACESGSMFEGILPNNIDVYATTAANTDEDSYGFYCPDLYPTPPPEYTTCLGDEYSISWLEDSDKNDMVNETLQQQYETVRRRTLVSHINATSHV 300
B Kang 57 MVIYMEACESGSMFEGILPNNIDVYATTAANTDEDSYGFYCPDLYPTPPPEYTTCLGDEYSISWLEDSDKNDMVNETLQQQYETVRRRTLVSHINATSHV 300
Rongxiandongdou MVIYMEACESGSMFEGILPNNIDVYATTAANTDEDSYGFYCPDLYPTPPPEYTTCLGDEYSISWLEDSDKNDMVNETLQQQYETVRRRTLVSHINATSHV 300
Nanxiadou 30 MVIYMEACESGSMFEGILPNNIDVYATTAANTDEDSYGFYCPDLYPTPPPEYTTCLGDEYSISWLEDSDKNDMVNETLQQQYETVRRRTLVSHINATSHV 300
Nandou 12 MVIYMEACESGSMFEGILPNNIDVYATTAANTDEDSYGFYCPDLYPTPPPEYTTCLGDEYSISWLEDSDKNDMVNETLQQQYETVRRRTLVSHINATSHV 300
Nanxiadou 25 MVIYMEACESGSMFEGILPNNIDVYATTAANTDEDSYGFYCPDLYPTPPPEYTTCLGDEYSISWLEDSDKNDMVNETLQQQYETVRRRTLVSHINATSHV 300
Willams 82 MQYGDKELNNDSLAIYIGALAPSLSLNENAHSFEQSTTQTKLISQRDTRLLHLRLELQKAQDGSEKLKAQKELADEIAHRKHVDNVVHLIGDLLFGEENS 400
Tongdou 11 MQYGDKELNNDSLAIYIGALAPSLSLNENAHSFEQSTTQTKLISQRDTRLLHLRLELQKAQDGSEKLKAQKELADEIAHRKHVDNVVHLIGDLLFGEENS 400
Shiyuehuang MQYGDKELNNDSLAIYIGALAPSLSLNENAHSFEQSTTQTKLISQRDTRLLHLRLELQKAQDGSEKLKAQKELADEIAHRKHVDNVVHLIGDLLFGEENS 400
B Kang 57 MQYGDKELNNDSLAIYIGALAPSLSLNENAHSFEQSTTQTKLISQRDTRLLHLRLELQKAQDGSEKLKAQKELADEIAHRKHVDNVVHLIGDLLFGEENS 400
Rongxiandongdou MQYGDKELNNDSLAIYIGALAPSLSLNENAHSFEQSTTQTKLISQRDTRLLHLRLELQKAQDGSEKLKAQKELADEIAHRKHVDNVVHLIGDLLFGEENS 400
Nanxiadou 30 MQYGDKELNNDSLAIYIGALAP..SLNENAHSFEQSTTQTKLISQRDARLLHLRLELQKAQDGSEKLKAQKELDDEIAHRKHIDNVVHLIGDLLFGEENS 398
Nandou 12 MQYGDKELNNDSLAIYIGALAP..SLNENAHSFEQSTTQTKLISQRDARLLHLRLELQKAQDGSEKLKAQKELDDEIAHRKHIDNVVHLIGDLLFGEENS 398
Nanxiadou 25 MQYGDKELNNDSLAIYIGALAP..SLNENAHSFEQSTTQTKLISQRDARLLHLRLELQKAQDGSEKLKAQKELDDEIAHRKHIDNVVHLIGDLLFGEENS 398
Willams 82 SAMMFHVRPAGKPLVDDWDCFKTLVKTYESQCGTLSSYGRKYTRAFANMCNAGIYEEQLKTTTSQACPQKNHA 473
Tongdou 11 SAMMFHVRPAGKPLVDDWDCFKTLVKTYESQCGTLSSYGRKYTRAFANMCNAGIYEEQLKTTTSQACPQKNHA 473
Shiyuehuang SAMMFHVRPAGKPLVDDWDCFKTLVKTYESQCGTLSSYGRKYTRAFANMCNAGIYEEQLKTTTSQACPQKNHA 473
B Kang 57 SAMMFHVRPAGKPLVDDWDCFKTLVKTYESQCGTLSSYGRKYTRAFANMCNAGIYEEQLKTTTSQACPQKNHA 473
Rongxiandongdou SAMMFHVRPAGKPLVDDWDCFKTLVKTYESQCGTLSSYGRKYTRAFANMCNAGIYEEQLKTTTSQACPQKNHA 473
Nanxiadou 30 SAMMFHVRPAGKPLVDDWDCFKTLVKTYESQCGTLSSYGRKYTRAFANMCNAGIYEEQLKTTSSQACPQKNHA 471
Nandou 12 SAMMFHVRPAGKPLVDDWDCFKTLVKTYESQCGTLSSYGRKYTRAFANMCNAGIYEEQLKTTSSQACPQKNHA 471
Nanxiadou 25 SAMMFHVRPAGKPLVDDWDCFKTLVKTYESQCGTLSSYGRKYTRAFANMCNAGIYEEQLKTTSSQACPQKNHA 471
