# Supplementary material for: Genetic mapping high protein content QTL from soybean ‘Nanxiadou 25’ and candidate gene analysis
Source: BMC Plant Biol. 2021 Aug 20;21:388. doi: 10.1186/s12870-021-03176-2 (PMC8377855; doi:10.1186/s12870-021-03176-2)
Supplement: Supplementary file 4 — Additional file 4: Figure S1. Location of quantitative trait loci (QTL) related to protein contents. For simplicity, only show the markers in the QTL confidence intervals, along with the terminal two markers at each end of the QTL-containing chromosomes [file 12870_2021_3176_MOESM4_ESM.pdf]

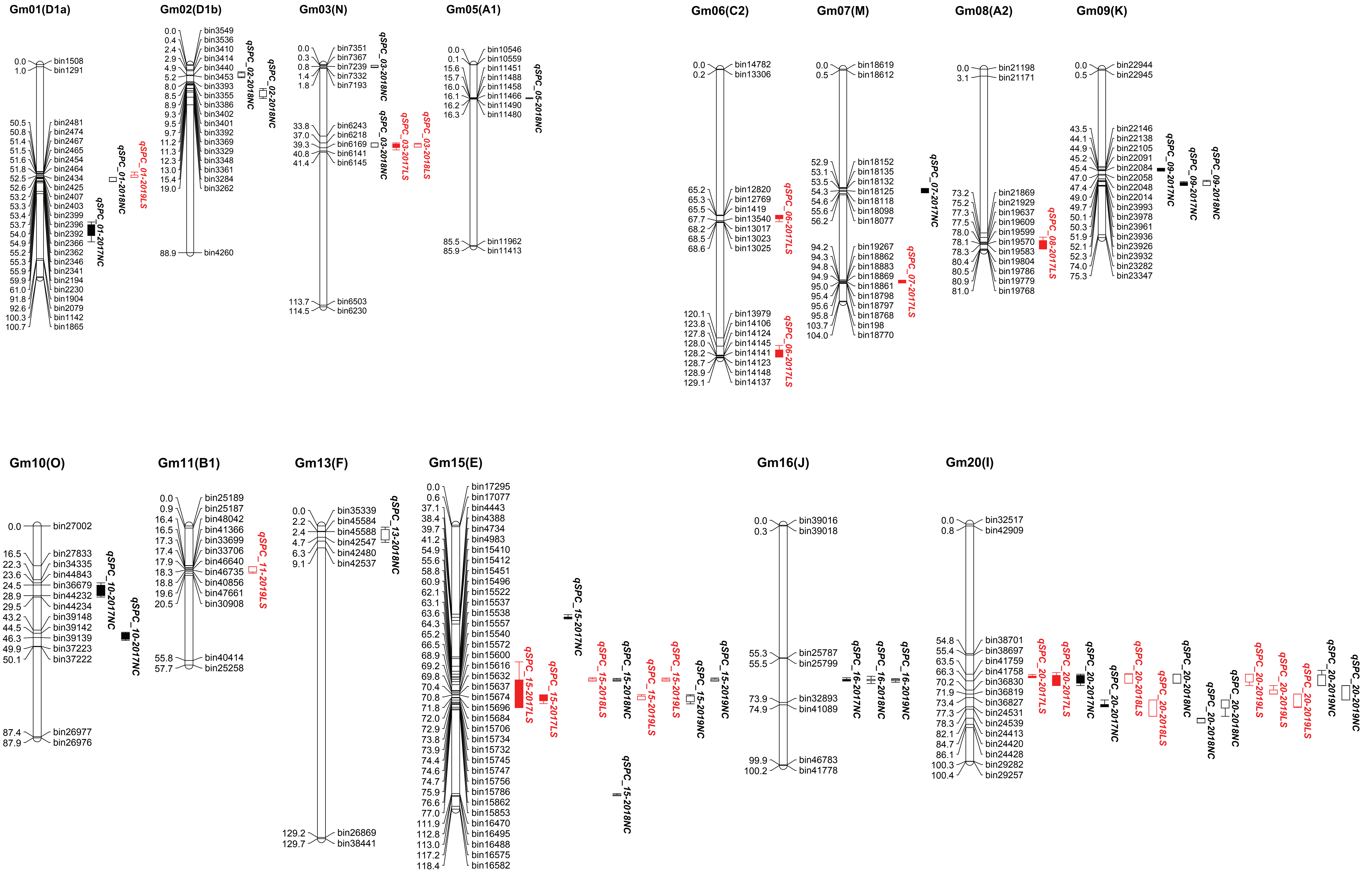
Gm01(D1a)
Gm02(D1b)
Gm03(N)
Gm05(A1)
Gm06(C2)
Gm07(M)
Gm08(A2)
Gm09(K)
Gm10(O)
Gm11(B1)
Gm13(F)
Gm15(E)
Gm16(J)
Gm20(I)
qSPC_01-2017NC
qSPC_01-2018NC
qSPC_01-2019LS
qSPC_02-2018NC
qSPC_03-2018NC
qSPC_03-2017LS
qSPC_03-2018LS
qSPC_05-2018NC
qSPC_06-2017LS
qSPC_07-2017NC
qSPC_07-2017LS
qSPC_08-2017LS
qSPC_09-2017NC
qSPC_09-2018NC
qSPC_10-2017NC
qSPC_11-2019LS
qSPC_13-2018NC
qSPC_15-2017NC
qSPC_15-2017LS
qSPC_15-2018LS
qSPC_15-2018NC
qSPC_15-2019LS
qSPC_15-2019NC
qSPC_16-2017NC
qSPC_16-2018NC
qSPC_16-2019NC
qSPC_20-2017LS
qSPC_20-2017NC
qSPC_20-2018LS
qSPC_20-2018NC
qSPC_20-2019LS
qSPC_20-2019NC
